# Supplementary material for: Quantifying the 60-Year Contribution of Japanese Zoos and Aquariums to Peer-Reviewed Scientific Research
Source: Animals (Basel). 2022 Feb 27;12(5):598. doi: 10.3390/ani12050598 (PMC8909568; doi:10.3390/ani12050598)
Supplement: Supplementary file 1 [file animals-12-00598-s001.zip › animals-1577032-supplementary.pdf]

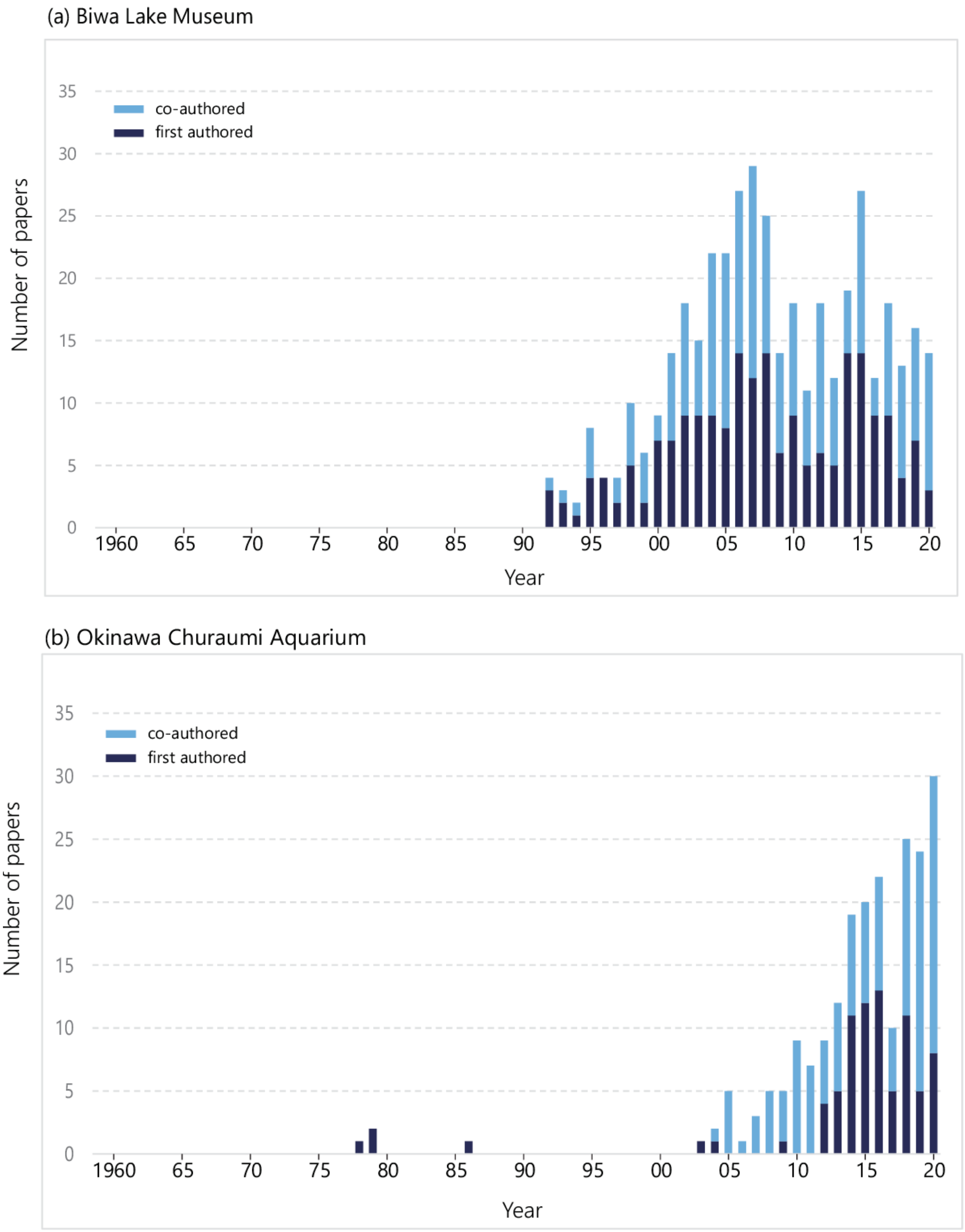

Figure S1. The annual trend of two aquariums in the number of papers coloerd by the afficiation of the first auther. Dark blue bars indicate each auariums staff as the first author, and light blue indicates outside researcher as one. (**a**) Biwa Lake Museum; (**b**) Okinawa Churaumi Aquarium.

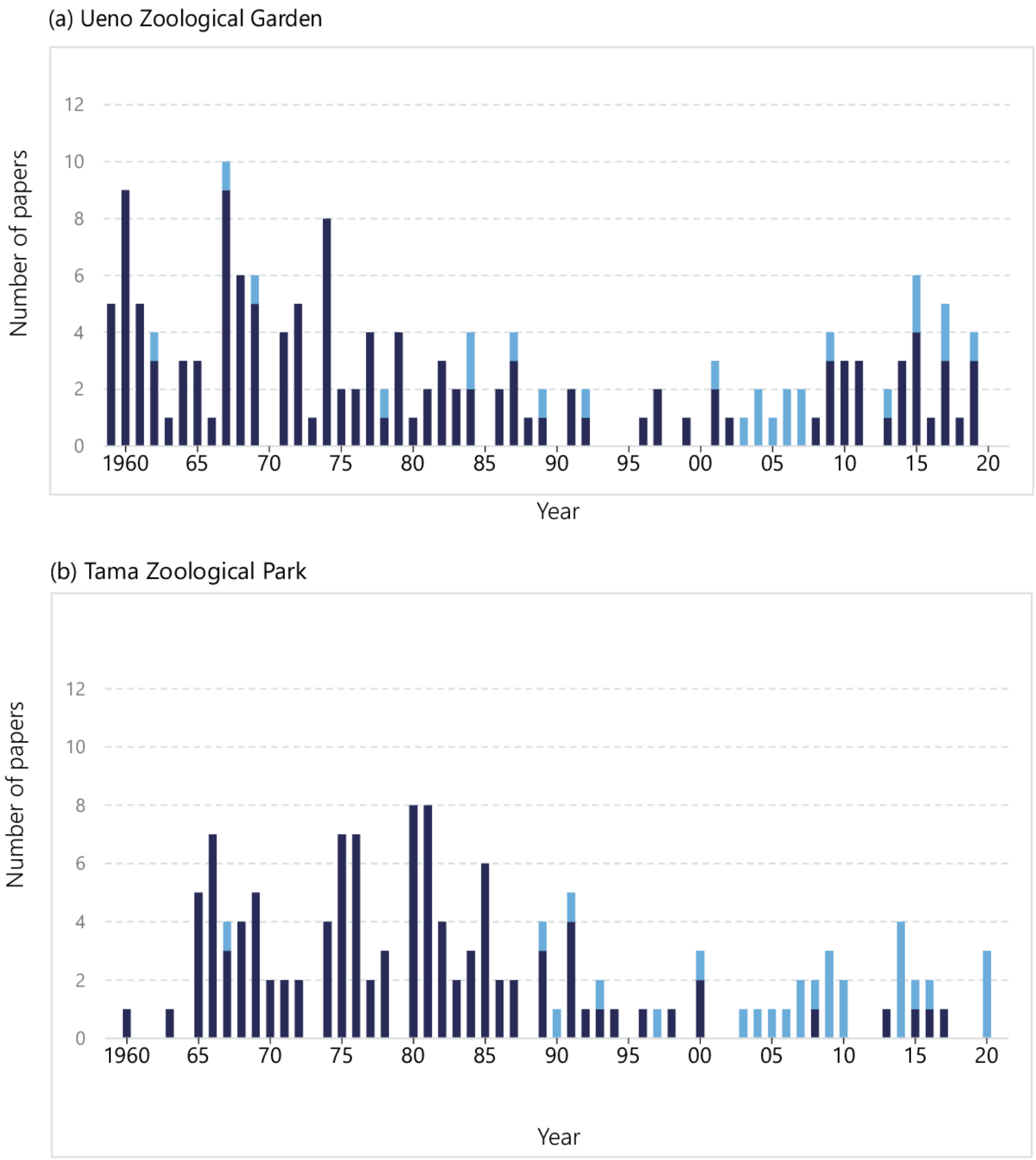

Figure S2. The annual trend of two zoos in the number of papers colored by the afficiation of the first auther. Dark blue bars indicate each zoo staff as the first author, and light blue indicates outside researcher as one. (**a**) Ueno Zoological Garden; (**b**) Tama Zoological Park.
